# Differential requirement for MEK Partner 1 in DU145 prostate cancer cell migration

**DOI:** 10.1186/1478-811X-7-26

**Published:** 2009-11-23

**Authors:** Electa R Park, Ashok K Pullikuth, Evangeline M Bailey, Donald E Mercante, Andrew D Catling

**Affiliations:** 1 Department of Biochemistry and Molecular Biology, LSU Health Sciences Center-New Orleans, LA, USA; 2 Department of Pharmacology, LSU Health Sciences Center-New Orleans, LA, USA; 3 Biostatistics Program, School of Public Health, LSU Health Sciences Center-New Orleans, LA, USA; 4 Stanley S Scott Cancer Center, LSU Health Sciences Center-New Orleans, LA, USA; 5 Laboratory of Integrin Signaling and Tumorigenesis, Van Andel Research Institute, Grand Rapids, MI, USA

## Abstract

ERK signaling regulates focal adhesion disassembly during cell movement, and increased ERK signaling frequently contributes to enhanced motility of human tumor cells. We previously found that the ERK scaffold MEK Partner 1 (MP1) is required for focal adhesion disassembly in fibroblasts. Here we test the hypothesis that MP1-dependent ERK signaling regulates motility of DU145 prostate cancer cells. We find that MP1 is required for motility on fibronectin, but not for motility stimulated by serum or EGF. Surprisingly, MP1 appears not to function through its known binding partners MEK1 or PAK1, suggesting the existence of a novel pathway by which MP1 can regulate motility on fibronectin. MP1 may function by regulating the stability or expression of paxillin, a key regulator of motility.

## Introduction

Prostate cancer is the most commonly diagnosed cancer in men. While *in situ *disease usually responds well to treatment, progression to an androgen-hypersensitive, metastatic state is often fatal [1]. Aberrant signaling through the ERK pathway correlates with progression in some models [2], although oncogenic mutations in Ras or Raf occur infrequently in prostate cancer [3,4]. Rather, prostate cancer commonly displays misregulation of cell-matrix adhesion signaling, including alterations in integrin expression and upregulation of Focal Adhesion Kinase (FAK) and p21-Activated Kinase (PAK) [5,6]. We have previously shown that PAK1 can activate MEK1 independently of Raf in model systems [7] suggesting that this alternative pathway to ERK activation may function in tumor cells lacking oncogenic mutations in Ras and Raf. Indeed, active PAK1 has been shown to stimulate ERK-dependent proliferation of breast cancer cells [8], and is constitutively active in DU145 prostate cancer cells [9].

MEK Partner 1 (MP1) is a putative scaffolding protein that binds MEK1 and ERK [10]. MP1 also binds to the late endosomal protein p14 [11], and the p14-MP1-MEK1 complex regulates trafficking and degradation of the EGFR [12]. Thus MP1/p14 provides a means of colocalizing the endocytosed EGFR and its downstream effectors during proliferation stimulated by EGF. MP1 and p14 also play important roles in cell morphogenesis and migration. MP1 and/or p14 are required for efficient activation of MEK and ERK, and control focal adhesion and actin dynamics during adhesion to fibronectin [13]. Since ERK is known to regulate focal adhesion disassembly [14,15], these observations implicate MP1-scaffolded ERK in focal adhesion dynamics. However, MP1 also binds active PAK1 [13], itself a regulator of adhesion disassembly [13,16,17], consistent with alternative mechanisms for its involvement in the regulation of cytoskeletal and adhesion dynamics [13]. Elevated PAK activity is associated with altered growth and migratory properties in a number of tumor types, including prostate cancer [18]. We therefore investigated whether MP1 influences ERK and/or PAK signaling during migration of DU145 prostate cancer cells.

## Materials and methods

### Reagents

EGF (Invitrogen) and UO126 were from Calbiochem. Gridded coverslips were from Bellco; glass-bottomed 35 mm microwell dishes were from MatTek Cultureware. Both were coated with fibronectin as described [19].

### Cell Culture

DU145 were maintained in RPMI-1640 containing 10% fetal bovine serum (FBS, Invitrogen).

### siRNA Assays

Cells (4.5 × 10^5 ^cells/100 mm dish plated two days prior to transfection) were transfected with 100 nM control siRNA (AGAGAGUAGUAGAGACAAUCGUCUG), MP1 Stealth siRNA (CAACACAGGACUAAUUGUCAGCCUA) or p14 Stealth siRNA (UUAAGAUGCCGCCACUUGGGUGAGG) (Invitrogen) using Lipofectamine 2000 (Invitrogen) according to manufacturer's instructions. Experiments were performed 72 hours post-transfection.

### Immunoblotting

Cells were typically lysed in RIPA buffer (50 mM Tris, 150 mM NaCl, 1% Triton X-100, 0.5% deoxycholate, 0.1% SDS, 5 mM EDTA, 50 mM NaF) supplemented with 1 μg/mL Leupeptin, 3 mM Benzamidine, 10 nM MicrocystinLR, 100 μM orthovanadate, 5 mM NaPPi, and 1 mM PMSF. Equal amounts of lysate protein were used for immunoblots. Proteins were detected using the following antibodies: MEK1 phospho-S298 [19]; MP1 [13]; MEK1 phospho-S218/S222 (Sigma); phosphoERK1/2 (clone12D4; Upstate) ERK1/2 phospho-T202/Y204 (Cell Signaling); paxillin (Biosource); PAK1 N-20 (Santa Cruz); pS141 PAK1-3 (Invitrogen). p14 antibody #5933 was raised against the peptide sequence Ac-CLKAKAQALVQYLEEPLTQVAAS-OH and affinity purified using standard techniques. Blots were imaged on an LAS-1000 Plus Intelligent Dark Box (Fujifilm) using Image Reader software (Fujifilm); the same software was used for quantification of immunoblot results.

### Adhesion/EGF stimulation assay

Cells were plated on fibronectin essentially as described [13]. For EGF stimulation, cells were washed in serum-free (SF) medium then fed SF medium for 2 hours prior to stimulation.

### Migration assays

Cells were trypsinized and collected in SF medium containing 1 mg/mL soybean trypsin inhibitor (Sigma), pelleted, suspended in SF medium and incubated at 37°C for two hours. 2 × 10^6 ^cells were allowed to form confluent monolayers on fibronectin-coated grid-etched coverslips for 5-7 hours at 37°C. Cells were treated with 1 μg/mL Mitomycin C (Sigma) and either 25 μM UO126 or DMSO vehicle for an additional 3 hours. The medium was aspirated and the coverslip was scratched 4 times with a 200 μL tip to form a # wound pattern. Loose cells were removed with SF medium; cells were fed fresh SF medium containing 1 μg/mL of Mitomycin C and either 25 μM UO126 or the equivalent volume of DMSO, with or without 1% FBS or 10 ng/mL EGF as indicated. Wounds were photographed in 6 different locations at zero-hour time point, and then incubated at 37°C for 5 hours (transfected cells) or 10 hours (untransfected cells) at which time the same locations were photographed. All photographs were taken with an Olympus IX81 microscope equipped with Slidebook software (Intelligent Imaging Innovations) and the area covered by migrating cells was calculated using ImageJ software (NIH). Parallel cultures were processed for immunoblotting.

### Immunofluorescence

Immunofluorescence was carried out essentially as described [13] using a Leica DMRA2 microscope equipped with Slidebook software. The number and size of vinculin-positive focal adhesions at the wound edge was calculated using Slidebook software.

### Kymography

2 × 10^6 ^cells were plated on 35 mm FN-coated glass-bottomed Petri dishes in SF medium with 1 μg/mL Mitomycin C and allowed to adhere for 5-7 hours. Confluent monolayers were wounded and imaged starting 10-20 min post wounding and again after 2 hours of migration. All cells were imaged for 5 minutes at 1 frame per second on an Olympus IX81 microscope equipped with Slidebook software. Kymographs were generated using ImageJ software.

### Statistical Analysis

Wound-healing assays were repeated in triplicate and statistical analysis was performed on the pooled results. A general linear model was constructed and linear contrast was used to perform pairwise comparisons of the means for the combined data set. Tukey's Honestly Significant test was used for post-hoc pairwise comparisons of migration under experimental conditions to that of migration under SF conditions and to each other. ANOVA on pooled data indicated significant differences within the data set.

## Results

### ERK activity is required for growth factor-, but not fibronectin-stimulated, DU145 migration

**Figure 1:**
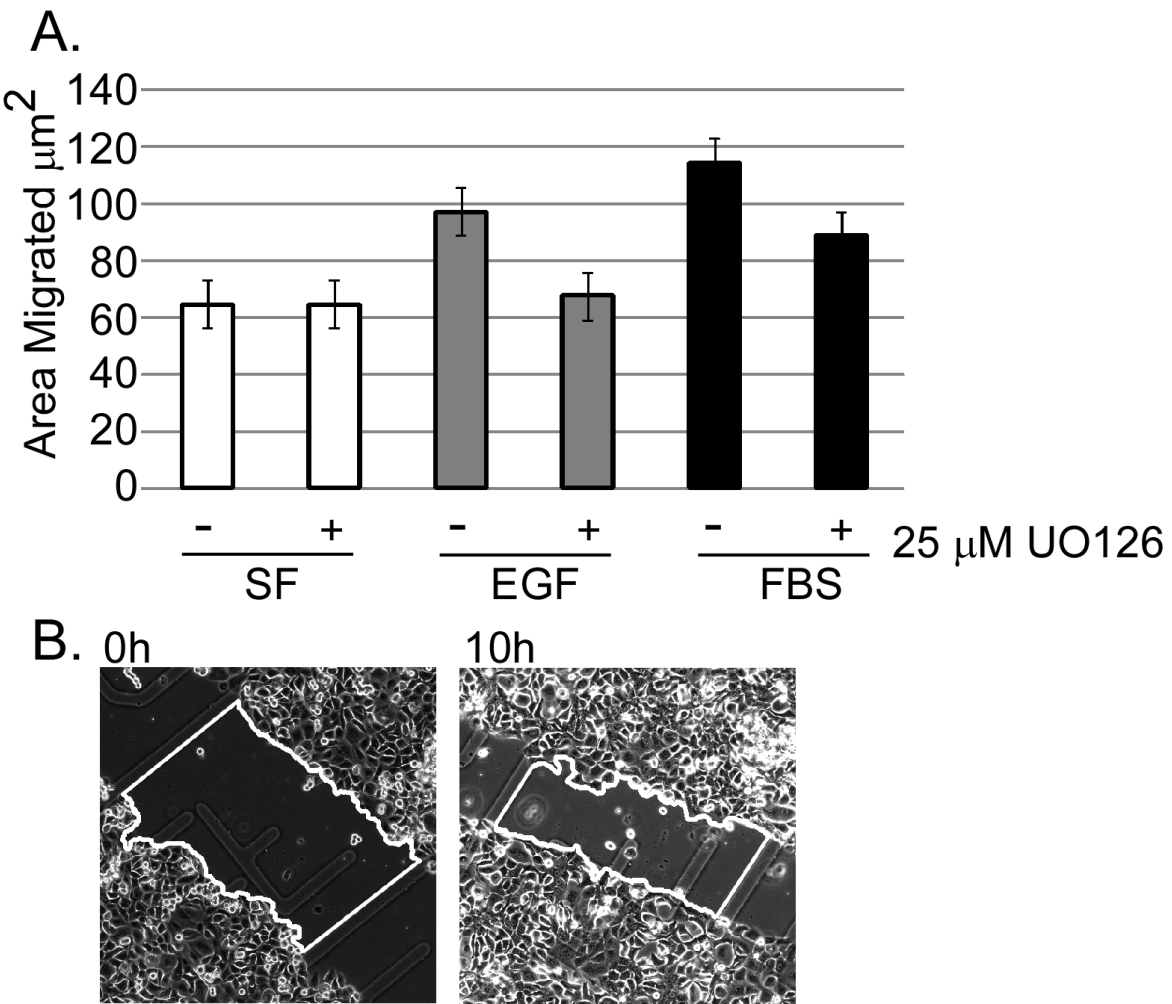
**ERK activation is not required for DU145 migration on FN, but is required for migration on FN induced by serum**. A) Results of DU145 wound healing on FN. Shown are averaged results from 4 experiments; error bars represent standard error of the mean (S.E.M.). Migration in response to fibronectin alone was not affected by UO126. Serum and EGF stimulated significant (p < 0.0001) increases in migration, that were significantly inhibited (p < 0.0001 for serum, p = 0.0004 for EGF) by UO126 treatment. B) Sample images from wound healing assay; for each condition, six grid squares were imaged at 0 and 10 h post-wounding, and wound area (outlined in white) was measured using ImageJ software (NIH).

We developed a quantitative wound-healing assay that makes use of gridded coverslips to allow for accurate measurement of migration into a wounded monolayer over time (Fig. 1B; Materials and Methods). To distinguish between wound closure due to cell movement and proliferation, mitosis in these assays was inhibited by the inclusion of 1 μg/mL mitomycin C; this concentration was determined as the minimal dose needed to prevent DU145 proliferation without inducing cell death under serum-free conditions (data not shown). Additionally, to distinguish between cell spreading and motility, cells were tracked in real time over the course of the assay (data not shown), and movies were analyzed for directionality and distance migrated. From these assays we were able to determine that DU145 are migrating into the wound in the monolayer over the timecourse of the assay. DU145 cells migrated 65 ± 3.5 μm^2 ^on fibronectin in serum-free conditions, and pre-treatment with 25 μM UO126 for 3 hr had no effect (Fig. 1A). Migration on fibronectin was increased to 115 ± 4.9 and 100 ± 5.3 μm^2 ^in the presence of 1% serum or 10 ng/mL EGF, respectively, and these increases were significantly inhibited by UO126 (Fig. 1A). Thus, MEK signaling is required for growth-factor-induced, but not for fibronectin-stimulated, migration of DU145 cells.

### MP1/P14 is required for migration of DU145 cells on fibronectin

**Figure 2:**
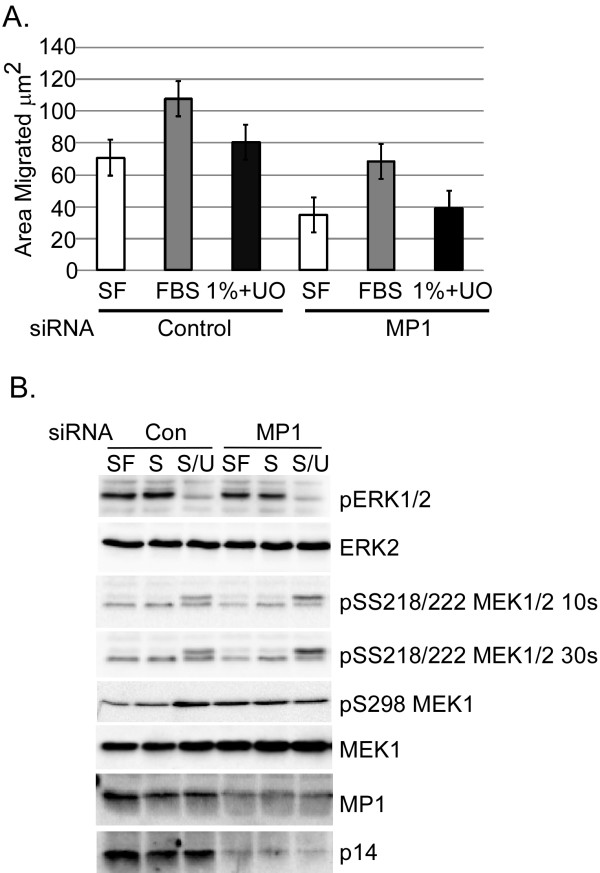
**MP1 knockdown inhibits migration in DU145 acutely adherent on fibronectin**. A) DU145 cells were transfected with Control (non-targeting) or MP1 siRNA. MP1 depletion inhibits DU145 migration on FN in serum-free medium (**SF**) by ~50% relative to control. Migration is induced by 1% fetal bovine serum (**1%**) in cells transfected with control or MP1 siRNA, and the induced component of migration is sensitive to MEK1 inhibition by UO126 (**UO**). Shown are mean data from three independent experiments; error bars represents S.E.M. B) Representative blot showing MP1 knockdown in DU145 cells treated in parallel with cells in wound-healing assay. Two exposures of MEK1 phospho-SS218/222 are shown to emphasize the low level of MEK1 activation in these assays, which nevertheless remains similar between Control and MP1 siRNA transfected cells. **SF**, **S**erum-**F**ree medium; **S**, 1% fetal bovine **S**erum medium; **S/U**, 1% fetal bovine **S**erum medium + 25 μM **U**O126.

Since fibronectin-stimulated motility of DU145 cells is insensitive to MEK inhibitor (Fig. 1A), we predicted that MP1 depletion would be similarly without effect on fibronectin-stimulated DU145 migration. However, since MEK activity is required for growth-factor stimulated DU145 motility (Fig. 1A), and MP1 is required for EGF- and serum-stimulated MEK/ERK activation in fibroblasts [13,20], we predicted that MP1 depletion would inhibit growth-factor stimulated motility of DU145 cells. Surprisingly, we found that fibronectin-stimulated motility was inhibited by ~50% following transfection with MP1 siRNA (Fig. 2A, white bars). In contrast, the component of motility stimulated by 1% serum (above and beyond that stimulated by fibronectin alone) was unaffected by transfection of MP1 siRNA, but was substantially inhibited by UO126 (Fig. 2A) as in naïve cultures (Fig. 1A). Consistent with these observations, depletion of MP1/p14 did not substantially inhibit levels of active ERK or active MEK in cells on fibronectin, whether or not they were exposed to growth factor (Fig. 2B), whereas UO126 essentially eliminated ERK activity in both control and MP1 siRNA-transfected cells (Fig. 2B). Note that knock-down of MP1 is difficult to measure accurately due to the presence of closely migrating proteins that cross react with our antibody. However, siRNAs targeting MP1 have been shown to cause a simultaneous reduction in p14 expression, and vice versa [13,21], most probably because the MP1-p14 heterodimer is more stable than the individual subunits. Since our p14 antiserum blots more cleanly, we used p14 levels as a surrogate measure for MP1 knockdown. p14 levels were reduced on average by 82% (Fig 2B; data not shown). Together, these data indicate that MP1/p14 but not MEK signaling is required for fibronectin-stimulated motility of DU145 cells, whereas MEK signaling but not MP1/p14 is required for motility stimulated by serum growth factors. Thus MP1/p14 and MEK play separable roles in DU145 migration.

### MP1 depletion decreases membrane activity and peripheral focal adhesions in migrating cells

**Figure 3:**
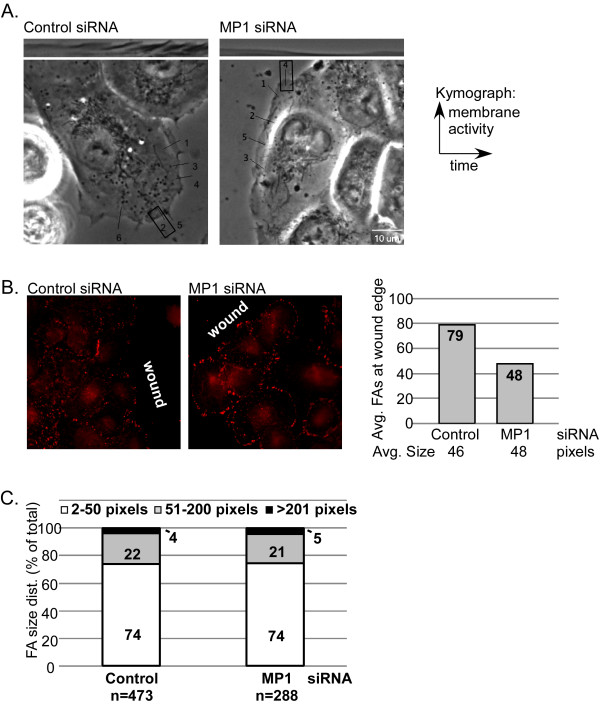
**MP1 depletion decreases membrane activity and the number of focal adhesions at the wound edge**. A) DU145 cells transfected with control or MP1 siRNA were wounded as described. Approximately 2 hours post-wounding, cells were imaged at 1 second intervals for 5 min for kymograph analysis. For each cell, 6-7 kymographs were generated (shown by numbered lines); 5-7 cells per condition were analyzed. Boxes around the number indicate the representative kymograph shown. B) Control and MP1 siRNA transfected cells plated on coverslips and imaged for a wound-healing assay were fixed and stained for vinculin to mark focal adhesions. Average number and size of FA occurring at the wound edge were quantified using ImageJ (chart). C) Size distribution of all focal adhesions in the cells imaged for B. MP1 depleted cells have fewer focal adhesions at the wound edge.

We next asked whether membrane protrusion or focal adhesions were altered in DU145 cells lacking MP1/p14. Kymograph analysis allows visualization of the membrane boundary over time; the y-value is determined by the position of the membrane edge, and the x-axis represents time [21,22]. Cells in MP1 siRNA-transfected cultures displayed much less protrusive and ruffling activity at the wound edge relative to control siRNA-transfected cells (Fig. 3A).

To examine focal adhesions in these assays, a monolayer was wounded and fixed after cells had migrated for 5 hrs. Control- and MP1 siRNA-transfected cells display adhesions of similar total number and size distribution (Fig. 3B, C). However, MP1 siRNA-transfected cells have ~50% fewer adhesions at the wound edge (Fig. 3B; defined as adhesions occurring between the cell periphery and the cortical actin ring, not shown; vinculin spots occurring at sites of cell-cell contact were not counted as focal adhesions). This suggests that early nucleation events required for adhesion formation at the wound edge occur at a lower frequency in cells depleted of MP1/p14, or that turnover of this population of adhesions is more pronounced in cells lacking MP1/p14.

### Depletion of MP1/p14 modestly inhibits PAK activity

**Figure 4:**
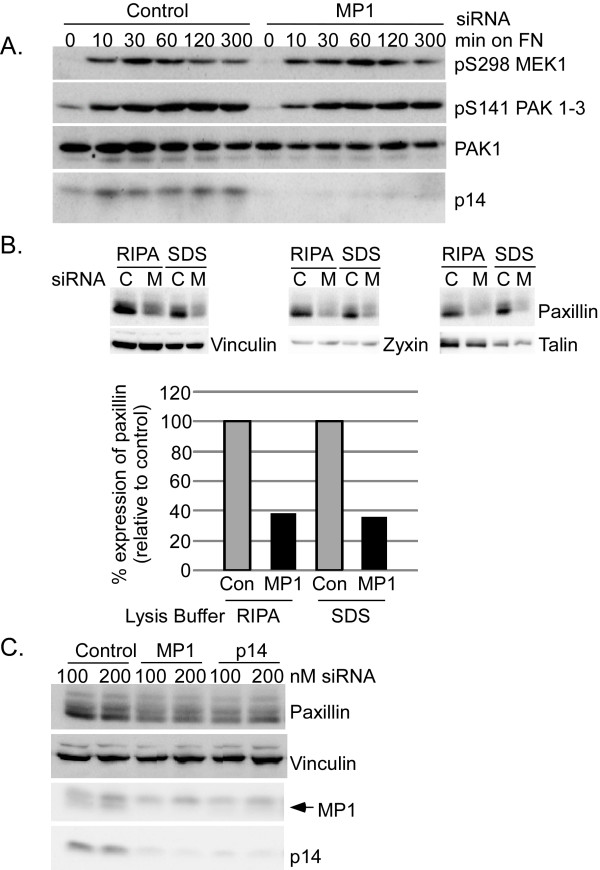
**MP1 knockdown modestly delays PAK autophosphorylation in response to FN and decreases paxillin expression**. A) DU145 cells transfected with control or MP1 siRNA were plated on FN for the times indicated. Whole cell lysates were analyzed by immunoblot for PAK phosphorylation of MEK1 (pS298 MEK1) and PAK1-3 autophosphroylation on S141. B) Cells transfected with control or MP1 siRNA were lysed in RIPA (RIPA) or SDS-PAGE (SDS) loading buffer (see Methods) and expression of focal adhesion proteins was assessed by immunoblot. C, Control siRNA; M, MP1 siRNA. Paxillin expression was quantified by densitometry and is expressed as percent of control for each lysis buffer. C) Cells transfected with control, MP1, or p14 siRNA were analyzed for vinculin, paxillin, MP1 and p14 expression.

Membrane protrusion and ruffling are governed in part by localized Rac/PI3-Kinase signaling, while membrane extensions are stabilized by focal complexes that also require PAK activity [16,17]. Focal adhesion maturation and disassembly are complex processes governed by GTPases (including Rac and Rho), kinases (including PAK, FAK, and ERK) and molecular organizing components including paxillin. Since fibronectin-stimulated DU145 motility was insensitive to MEK inhibitor but sensitive to knock-down of MP1/p14, we reasoned that MP1 functions through a MEK activity-independent pathway to regulate membrane and adhesion dynamics. One candidate molecule is PAK1, to which MP1 binds [13]. Group I PAK kinases phosphorylate a number of cellular targets, including MEK1 [7,19,23], paxillin [24], and themselves [25,26]. Depletion of MP1/p14 slightly delays autophosphorylation of PAK1-3 on S141 following adhesion to fibronectin, with phosphorylation recovering around 1 hr and persisting throughout the 5 hr time course. Maximal phosphorylation of MEK1 on S298 is similarly delayed in MP1 siRNA-transfected cells (Fig. 4A). While we cannot exclude that these subtle differences in kinetics of phosphorylation in the population as a whole represent significant quantitative differences at the wound edge, our data suggest that depletion of MP1/p14 has minor effects on the overall activity of the PAKs phosphorylating these substrates.

### Paxillin is largely absent in DU145 cells depleted of MP1/p14

To extend these observations, we asked whether PAK phosphorylation of the cytoskeletal linker/adapter paxillin was regulated by MP1/p14. Paxillin has been shown to play essential roles in focal adhesion dynamics, membrane protrusion and motility [14,27-29]. PAK phosphorylates paxillin on S273, and phosphorylation of this site is required for stabilizing membrane protrusion [24]. Surprisingly, the abundance of paxillin was substantially reduced in cells depleted of MP1/p14 (~60% reduction, Fig. 4B), suggesting that paxillin is poorly expressed or unstable in cells lacking MP1/p14, or that it resides in a compartment not extracted by our lysis buffer. To exclude the latter possibility, we prepared extracts in SDS-PAGE sample buffer which is expected to solubilize all cellular proteins. The abundance of paxillin was similarly reduced (by ~60%) in SDS-PAGE buffer extracts of DU145 cells transfected with MP1 siRNA, while expression of other focal adhesion markers vinculin, talin and zyxin was unaffected by knock-down of MP1/p14 (Fig. 4B). Reduction of paxillin levels is unlikely to result from off-target effects of the MP1 siRNA since similar results were obtained with an independent p14 siRNA (Fig. 4C). These data indicate that the MP1 and/or p14 are required to maintain normal paxillin protein levels.

## Discussion

### MP1/p14 regulated signaling events

MP1/p14 is essential for ERK activation during adhesion of fibroblasts to fibronectin, and in this context, depletion of MP1/p14 causes an apparent stabilization of focal adhesions [13]. We therefore predicted that depletion of MP1/p14 would cause inhibition of fibronectin-stimulated ERK activation and stabilization of focal adhesions in DU145 cells, and thus inhibit cell motility. To our surprise, we found that migration of DU145 cells on fibronectin does not require acute MEK activity but does require MP1/p14. Conversely, migration stimulated by growth factors requires acute MEK activity, but does not require MP1/p14. Furthermore, MP1/p14 depletion does not significantly inhibit ERK phosphorylation in response to fibronectin or growth factor stimulation. These data indicate that there are separable MEK activity- and MP1/p14-dependent pathways leading to DU145 motility in response to serum growth factors and fibronectin respectively.

Because Group I PAKs have multiple roles in cell motility [30] and PAK1 binds MP1 [13], we reasoned that MP1 might function through PAK to regulate fibronectin-stimulated DU145 migration. However, depletion of MP1 caused only modest differences in PAK autophosphorylation or PAK phosphorylation of MEK1; phosphorylation of the PAK-dependent substrate cofilin was also unaffected by depletion of MP1 (data not shown). These data suggest that PAK is not a major effector for MP1 in DU145 cell motility.

### MP1/p14 is required for maintenance of paxillin levels

Surprisingly, we found that paxillin was largely absent from DU145 cells depleted of MP1/P14 while expression of other focal adhesion proteins was unaffected. Future experiments will determine whether this loss of paxillin results from decreased mRNA or protein synthesis, or increased turnover of paxillin protein.

### Depletion of MP1/p14 influences membrane dynamics and focal adhesion abundance

We have found that depletion of MP1/p14 results in a loss of paxillin, decreases membrane protrusion, and reduces the number of focal adhesions at the wound edge. Paxillin serves as a signaling nexus at focal adhesions and in the leading edge of migrating cells. In particular, the GIT1-PIX-PAK complex is stabilized by PAK phosphorylation of paxillin, leading to increased localized Rac activation, membrane protrusion, and migration [24]. This complex localizes to a population of dynamic focal complexes at the leading edge [14,24], suggesting that it may be important for stabilizing membrane protrusion. Alternatively, paxillin may liberate p190A RhoGAP from p120 RasGAP at the leading edge of motile cells [31]. Locally released p190A RhoGAP is free to stimulate hydrolysis of GTP on Rho allowing membrane protrusion. Interestingly, while the loss of paxillin correlates with a failure of DU145 to migrate on fibronectin, it does not preclude serum-stimulated motility.

Our data are the first to indicate that MP1/P14 has important functional roles outside the ERK pathway. These observations suggest that MP1/p14- and paxillin-dependent and -independent pathways for motility may be operational in DU145 cells in a context-specific manner.

## Competing interests

The authors declare that they have no competing interests.

## Authors' contributions

ERP carried out the migration and biochemical assays and drafted the manuscript; AKP provided technical expertise for the microscopy and for quantitative analysis of migration, kymography and focal adhesion size and abundance; EMB performed knock-down experiments investigating paxillin abundance; DEM performed statistical analysis for the migration assays; and ADC was responsible for the overall design and coordination of the study. All authors read and approved the final manuscript.
